# Aberrant gene expression in mucosa adjacent to tumor reveals a molecular crosstalk in colon cancer

**DOI:** 10.1186/1476-4598-13-46

**Published:** 2014-03-05

**Authors:** Rebeca Sanz-Pamplona, Antoni Berenguer, David Cordero, David G Molleví, Marta Crous-Bou, Xavier Sole, Laia Paré-Brunet, Elisabet Guino, Ramón Salazar, Cristina Santos, Javier de Oca, Xavier Sanjuan, Francisco Rodriguez-Moranta, Victor Moreno

**Affiliations:** 1 Unit of Biomarkers and Susceptibility, Catalan Institute of Oncology (ICO), Bellvitge Biomedical Research Institute (IDIBELL) and CIBERESP, L’Hospitalet de Llobregat, Barcelona, Spain; 2 Translational Research Lab, Catalan Institute of Oncology (ICO), Bellvitge Biomedical Research Institute (IDIBELL), L’Hospitalet de Llobregat, Barcelona, Spain; 3 Medical Oncology Service, Catalan Institute of Oncology (ICO), Bellvitge Biomedical Research Institute (IDIBELL), L’Hospitalet de Llobregat, Barcelona, Spain; 4 General and Digestive Surgery Service, University Hospital Bellvitge (HUB–IDIBELL), L’Hospitalet de Llobregat, Barcelona, Spain; 5 Department of Clinical Sciences, Faculty of Medicine, University of Barcelona (UB), Av. Gran Vía 199-203, 08908 L’Hospitalet de Llobregat, Barcelona, Spain; 6 Pathology Service, University Hospital Bellvitge (HUB–IDIBELL), Barcelona, L’Hospitalet de Llobregat, Spain; 7 Department of Gastroenterology, University Hospital Bellvitge (HUB–IDIBELL), L’Hospitalet de Llobregat, Barcelona, Spain

**Keywords:** Colorectal cancer, Network, Microenvironment, Molecular crosstalk, Systems biology

## Abstract

**Background:**

A colorectal tumor is not an isolated entity growing in a restricted location of the body. The patient’s gut environment constitutes the framework where the tumor evolves and this relationship promotes and includes a complex and tight correlation of the tumor with inflammation, blood vessels formation, nutrition, and gut microbiome composition. The tumor influence in the environment could both promote an anti-tumor or a pro-tumor response.

**Methods:**

A set of 98 paired adjacent mucosa and tumor tissues from colorectal cancer (CRC) patients and 50 colon mucosa from healthy donors (246 samples in total) were included in this work. RNA extracted from each sample was hybridized in Affymetrix chips Human Genome U219. Functional relationships between genes were inferred by means of systems biology using both transcriptional regulation networks (ARACNe algorithm) and protein-protein interaction networks (BIANA software).

**Results:**

Here we report a transcriptomic analysis revealing a number of genes activated in adjacent mucosa from CRC patients, not activated in mucosa from healthy donors. A functional analysis of these genes suggested that this active reaction of the adjacent mucosa was related to the presence of the tumor. Transcriptional and protein-interaction networks were used to further elucidate this response of normal gut in front of the tumor, revealing a crosstalk between proteins secreted by the tumor and receptors activated in the adjacent colon tissue; and vice versa. Remarkably, Slit family of proteins activated ROBO receptors in tumor whereas tumor-secreted proteins transduced a cellular signal finally activating AP-1 in adjacent tissue.

**Conclusions:**

The systems-level approach provides new insights into the micro-ecology of colorectal tumorogenesis. Disrupting this intricate molecular network of cell-cell communication and pro-inflammatory microenvironment could be a therapeutic target in CRC patients.

## Background

Colorectal cancer (CRC) is a complex disease in which many genes, proteins, and molecular processes are implicated. Proteins do not work independently in a tumor cell, but are organized into co-regulated units or pathways that perform a common biological function [1]. Relevant molecular mechanisms involved in cancer are gene regulation, signaling, cell metabolism, and the connections between them, among others [2]. In addition to the tumor cell intrinsic complexity, increasing data support the main role of tumor microenvironment in the mechanisms of CRC progression [3-5]. Tumor microenvironment is composed by a heterogeneous population of stromal cells such as fibroblasts and immune cells, extracellular matrix components and secreted factors. All these components work orchestrated by molecular transducers like integrins engaging cell-cell and cell-matrix signaling that in turn enhance tumor growth [6].

Besides, a colorectal tumor is not an isolated entity growing in a restricted location of the body. An active communication exists not only between different cell communities within the tumor bulk but also between the tumor and the non-tumor distant mucosa. Hence, the patient’s gut environment constitutes the framework where the tumor evolves and this relationship promotes and includes a complex and tight correlation with inflammation, blood vessels formation, nutrition and gut microbiome composition [7]. Consequently, studying the micro-ecology context of a tumor is central to understand colorectal carcinogenesis. The tumor influence on environment could both promote an anti-tumor and a pro-tumor response. Some microenvironments, particularly those associated with tissue injury, are favorable for progression of mutant cells, whereas others restrict it. Cancer cells can also instruct surrounding tissues to undergo changes that promote malignancy [8].

Field cancerization or the field-effect is a theory first described by Slaughter et al. in oral carcinoma [9]. In the initial phase of the multistep carcinogenesis, a stem cell acquires genetic alterations and forms a “patch”, a clonal unit of altered daughter cells. Further alterations convert the “patch” into a field of pre-neoplastic cells. Although only one cell becomes tumoral, the remaining field (adjacent mucosa) continues in a “pre-neoplastic-state” composed of morphologically normal, but biologically altered epithelial cells. Since this field is a pre-tumor site predisposed towards development of cancer, this hypothesis could explain local recurrences after surgery [10].

Understanding the complex ways in which cancer cells interact with their surroundings, both locally in the tumor organ and systemically in the body as a whole has implications for effective cancer prevention and therapy. In contrast to the gene-centric view, a systems biology approach (defined as the analysis of the molecular relationship between genes and proteins as a whole) can be useful to depict a global view of the cancer disease not only as a tumor cell but as an intricate systemic disease [11].

**Figure 1:**
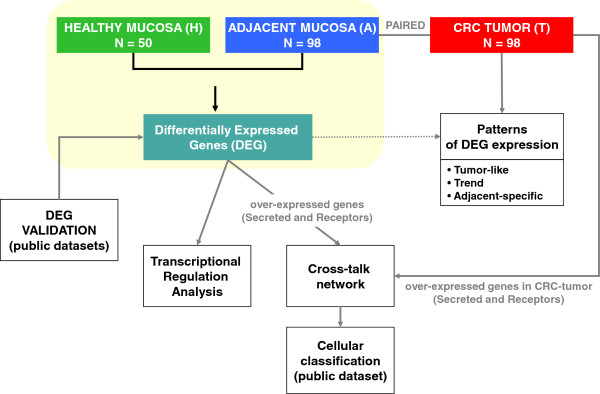
**Work flow chart.** The central core of the analysis is the comparison between adjacent mucosa and healthy mucosa at transcriptomic (gene expression data) and transcriptional (regulatory network) level. Independent public datasets were used to validate the results. In a second step, tumor tissue was used to search for different DEG patterns. Finally, a crosstalk network was inferred to decipher molecular communication between the tumor and the adjacent gut underlying DEG. Public data was used to elaborate a cellular classification of genes implicated in the crosstalk.

In this study, mRNA expression from paired tumor (T) and adjacent mucosa from CRC patients (A) and mRNA from mucosa healthy donors (H) were measured using microarrays. The inclusion of samples from healthy subjects has allowed us assessing whether adjacent mucosa from colon cancer patients differs from healthy donors’ mucosa possibly due to the tumor presence. Indeed, a number of differentially expressed genes (DEG) were found between these two entities (A vs. H). Considering their level of expression in tumor tissues, these DEGs were classified as “Tumor-like”, “Trend” or “Adjacent-specific” (A vs. T) patterns. To explain the mechanisms that regulate these patterns of differential expression, networks mimicking transcription regulation were used to search for those transcription factors directly influencing DEG. Then, a systems biology approach using PPIN was applied to describe a crosstalk between cytokines and other proteins secreted by the tumor and receptors activated in the adjacent colon tissue; and vice versa, providing new insights into the micro-ecology of colorectal tumorigenesis. Finally, relevant cytokines and receptors up-regulated in tumor tissue were identified comparing T vs. H expression (Figure 1). Further elucidation of these interactions could be helpful in the development of novel therapeutic strategies oriented to disrupt this molecular crosstalk.

## Results

### Characterization of differentially expressed genes between adjacent and healthy mucosa

**Figure 2:**
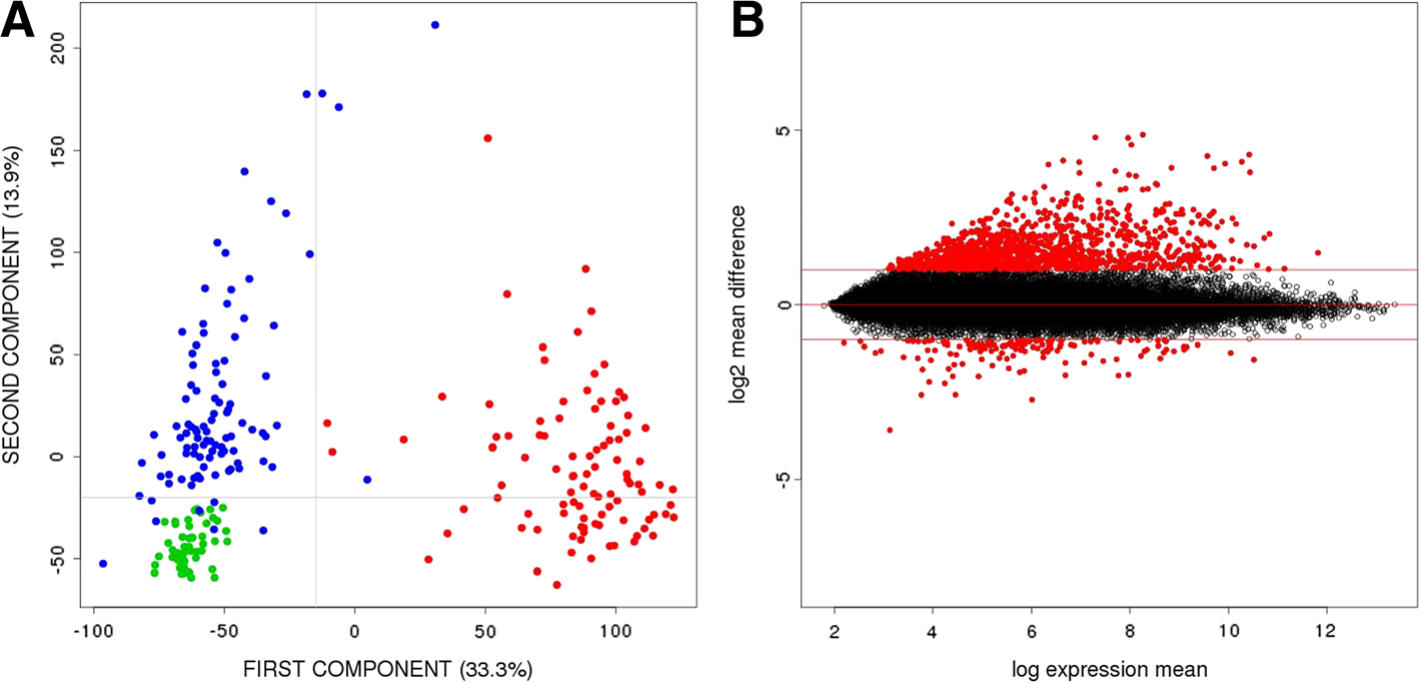
**Gene expression differences between adjacent and healthy mucosa samples. A**. PCA scatter plot representing the dispersion of the samples based on their gene expression levels. Tumor samples (red), adjacent mucosa samples (blue) and samples from healthy donors (green) were plotted in 1^st^ and 2^nd^ principal components. **B**. MA Plot representing gene expression differences between adjacent and healthy mucosa samples. In red, those probes with a FDR < 1% and log2 mean difference > 1.

A principal component analysis (PCA) was done to explore the variability of the transcriptomic data from our 246 samples (Figure 2A). As expected, tumor samples appeared as an independent cluster (T in red). Surprisingly, adjacent paired mucosa (A in blue) were also clearly separated from healthy mucosa (H in green), reflecting a large number of differentially expressed genes (DEG) between them. A total of 895 genes were differentially expressed at FDR < 1% and log2 mean difference > 1 between adjacent and healthy mucosa (Additional file 1: Table S1). Interestingly, 88% of these genes were over-expressed in adjacent mucosa (Figure 2B).

The functional enrichment analysis of these genes identified the classical pathways involved in cancer and were highlighted by a significant enrichment of functions related to *Inhibition of matrix metalloproteinases*, *Cell adhesion molecules*, *cytokine-cytokine receptor interaction*, *TGF-beta signaling pathway*, *integrin signaling pathway, complement and coagulation cascades*, *wound healing*, *response to external stimulus*, *inflammatory response* and *soluble fraction,* among others (see complete list in Additional file 2: Table S2, Additional file 3: Table S3 and Additional file 4: Figure S1). This functional analysis suggested an active reaction of the adjacent mucosa related to the presence of the tumor or a more passive reaction induced by factors released from the tumor.

Public transcriptomic data analyzing adjacent and healthy mucosa were used to validate the list of DEG. As a result, 60% of the genes were validated at FDR 1%. At FDR 5%, 91% of the genes were validated (Additional file 5: Table S4 and Additional file 4: Figure S2). These results should be interpreted with caution because each sample type was analyzed in different experiments and, though we normalized the data jointly, we cannot exclude strong batch or laboratory effects. We could not find a dataset like ours, in which healthy and adjacent colon mucosa were analyzed simultaneously.

**Figure 3:**
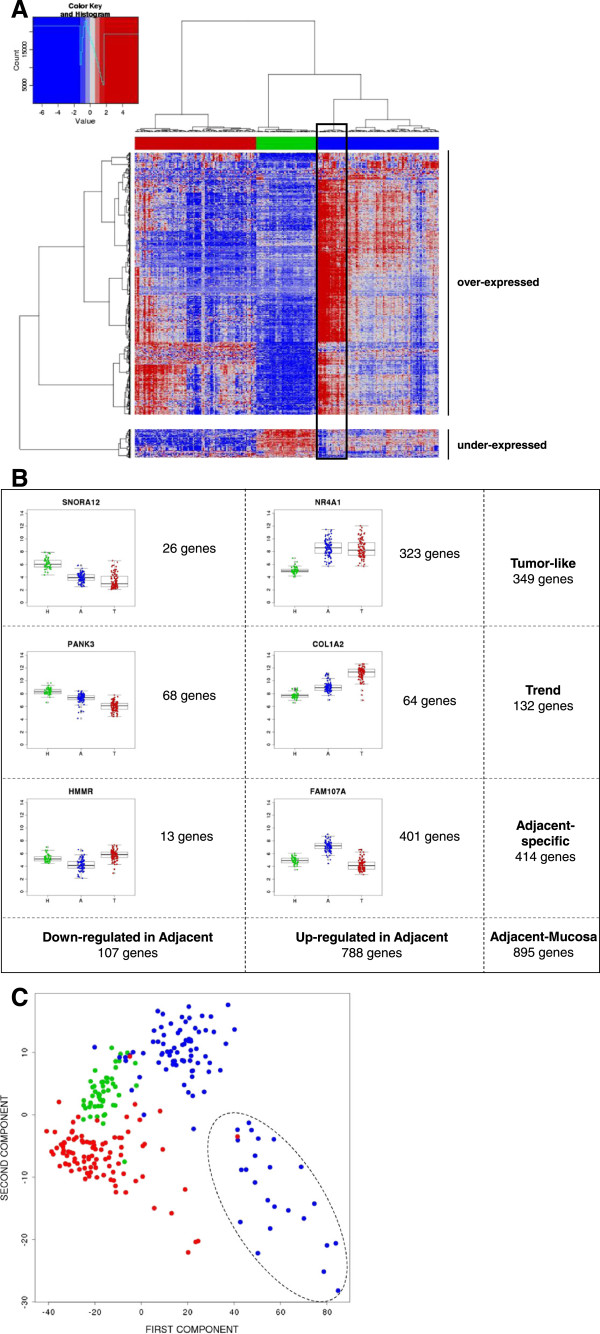
**DEG characterization. A**. Hierarchical clustering of 1230 over-expressed and 136 under-expressed probes that correspond to 788 and 107 genes respectively classifying the 246 tissue samples into three clusters of healthy mucosa (green), tumors (red) and adjacent mucosa (blue). Highlighted in black, the group of 24 adjacent samples showing an extreme phenotype. **B**. Representative DEG patterns are displayed. DEG between adjacent and mucosa were classified as “Tumor-like”, “Trend” and “Adjacent-specific” genes. **C**. PCA using “Adjacent-specific” DEG. Tumor samples (T) are painted in red, adjacent samples (A) in blue and healthy mucosa (H) in green. The 24 adjacent samples showing an extreme phenotype are circled with a dot line.

Figure 3A shows a hierarchical clustering performed with the set of DEG between adjacent mucosa (A) and healthy mucosa (H). Interestingly, the three different tissues were perfectly classified, including the tumors (T) that did not participate in the gene selection. Regarding genes, three patterns of expression were identified as shown in Figure 3B: a) “Tumor-like” (A = T > H or H > A = T) when genes in A had similar pattern as T (349 genes); b) “Trend” (T > A > H or H > A > T) when genes in A had an intermediate expression between H and T (132 genes); and c) “Adjacent-specific” (A < (T,H) or A > (T,H)), when genes were specifically de-regulated in A when compared to either T or H, irrespective of the relationship between T and H (414 genes). The size of this latter group was a surprise that lead us to explore in detail a crosstalk between the tumor and the adjacent mucosa.

Regarding enriched functions for these gene patterns, Tumor-like functions included *AP-1 transcription factor network*, *COX reactions* or *activation of AP-1*, whereas Adjacent-specific functions were enriched in *axon guidance, PPAR signaling pathway or BMP2 signaling pathway*, among others. These results suggest different functions for each gene expression pattern, though *Integrin signaling pathway*, *complement cascade*, *adhesion* or *Interferon signaling* were functions shared by the two patterns (see complete list in Additional file 6: Table S5).

Adjacent mucosa samples appeared divided into two groups in the hierarchical clustering analysis (Figure 3A). The smallest of them, with 24 samples, was characterized by high expression in most of adjacent-specific genes. A PCA performed with these adjacent-specific genes showed that the second component was capturing the specificity of this sample cluster and that adjacent mucosa were more similar to tumor than to healthy mucosa (Figure 3C). In fact, the original PCA analysis with all genes also identified these adjacent mucosa samples as highly variable in the second component (Figure 2A).

These clusters were not associated with the clinical parameters gender, age and tumor progression neither with technical parameters RNA integrity value (RIN), 260/230 ratio and plate. In addition, a functional analysis including differentially expressed genes between these two clusters did not show specific functions but essentially those described as characteristic of adjacent mucosa. These results suggest that the smaller cluster of adjacent samples was just an extreme phenotype of these samples. Interestingly, this pattern was also observed in the validation dataset (see heatmap in Additional file 4: Figure S2).

### Transcriptional regulation of differentially expressed genes between adjacent and healthy mucosa

We hypothesized that this differential expression could be triggered by a transcriptional program, activated only in adjacent mucosa by the presence of the tumor, and normally silenced in healthy mucosa. This hypothesis was supported by the GSEA results, in which 312 transcription factors motifs were found to be statistically associated with the adjacent mucosa phenotype (nominal p-value < 0.01) but none was found associated to healthy mucosa phenotype (Additional file 3: Table S3).

**Figure 4:**
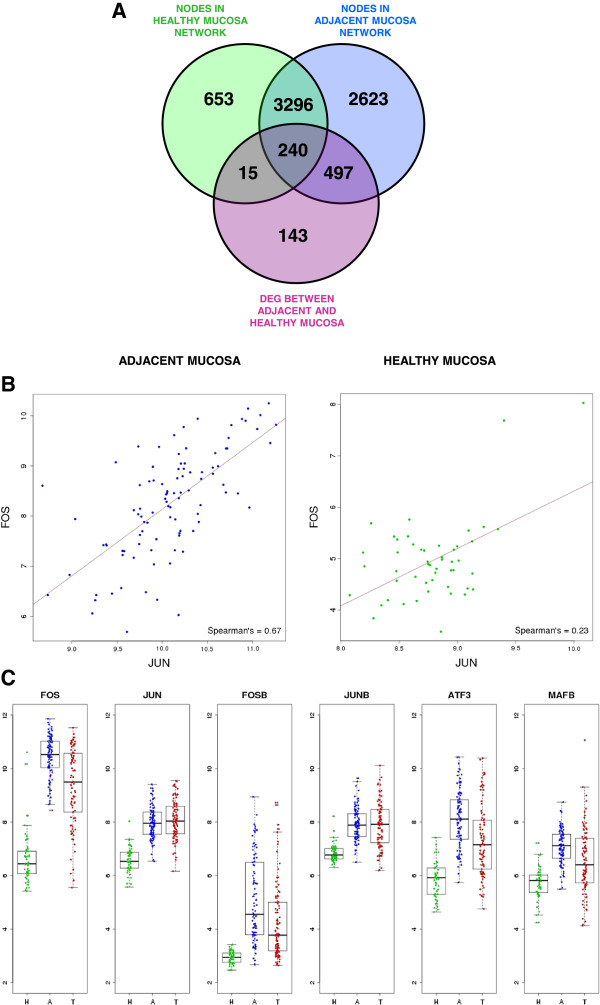
**DEG analysis in the framework of transcriptional networks. A**. Venn Diagram showing the overlap between nodes in adjacent mucosa transcriptional network (blue) and healthy mucosa transcriptional network (green). DEG were merged with the two transcriptional networks. **B**. Expression correlation between transcription factors Jun and Fos in adjacent (blue) and healthy mucosa (green). **C**. Gene expression levels of AP-1 subunits in healthy mucosa (green) adjacent mucosa (blue) and tumor tissue (red).

To further explore this hypothesis, transcriptional networks were inferred and compared using gene expression data of adjacent and healthy mucosa (see Additional file 4: Figure S3). Venn diagram in Figure 4A shows the overlap between nodes of each network. The vast majority of healthy mucosa nodes were also active in adjacent mucosa network whereas 3120 new nodes appeared specific to the adjacent mucosa and 668 nodes disappeared from the network. As expected, DEG between adjacent and healthy mucosa were over-represented in the new active nodes of the adjacent mucosa network (empirical p-value < 10^-4^) suggesting that DEG are not only performing common functions but also co-regulated in a sub-transcriptional network not active in healthy mucosa samples. Out of 895 DEG, 60 (13%) were transcription factors (TF), and random re-sampling of genes among the complete dataset revealed that DEG were significantly enriched in TF (empirical p-value < 0.001). Among these 60 TF, 35 were specific of the adjacent mucosa transcriptional network.

TF were ranked taking into account the total number of their targets (degree) and the proportion of targets in our DEG list. This rank suggested sub-networks specifically active in adjacent mucosa tissue. TF with higher rank were more specific of adjacent mucosa, and showed higher values of eccentricity (a topological network measure of the spreading of a node in the network) and lower values of closeness centrality (Table 1).

**Table 1:** List of transcription factors differentially expressed between adjacent and healthy mucosa samples

**Gene symbol**	**Connections**	**DEG connections**	**Rank**	**P-value**	**Eccentricity**	**Closenness centrality**	**Healthy mucosa network**
FOSB	4	4	1.000	<1.00E-04	9	0.209	NO
JUN	2	2	1.000	<1.00E-04	11	0.151	NO
NR4A2	5	5	1.000	<1.00E-04	9	0.204	NO
OSR2	12	12	1.000	<1.00E-04	8	0.234	NO
ZBTB16	1	1	1.000	<1.00E-04	9	0.200	NO
EGR2	10	9	0.900	<1.00E-04	8	0.247	NO
NR4A3	9	8	0.889	<1.00E-04	8	0.249	NO
EBF1	162	143	0.883	<1.00E-04	7	0.294	YES
HEY2	24	21	0.875	<1.00E-04	8	0.266	NO
NR4A1	16	14	0.875	<1.00E-04	8	0.240	NO
PRRX1	30	26	0.867	<1.00E-04	7	0.289	NO
EGR1	13	11	0.846	<1.00E-04	9	0.217	YES
FOS	6	5	0.833	<1.00E-04	10	0.178	NO
JUNB	5	4	0.800	<1.00E-04	10	0.178	NO
MEOX2	75	60	0.800	<1.00E-04	7	0.303	NO
ZFPM2	133	106	0.797	<1.00E-04	7	0.296	NO
ERG	76	60	0.789	<1.00E-04	8	0.287	NO
TSHZ2	109	85	0.780	<1.00E-04	7	0.302	NO
FOXC1	27	21	0.778	<1.00E-04	8	0.268	NO
HLF	84	65	0.774	<1.00E-04	7	0.301	NO
MEIS2	133	100	0.752	<1.00E-04	8	0.305	NO
CREB5	32	24	0.750	<1.00E-04	8	0.278	NO
PRDM6	83	62	0.747	<1.00E-04	7	0.293	NO
GLIS2	79	58	0.734	<1.00E-04	7	0.302	NO
HAND2	138	101	0.732	<1.00E-04	7	0.289	NO
EGR3	11	8	0.727	<1.00E-04	8	0.263	NO
SOX18	40	29	0.725	<1.00E-04	7	0.274	NO
ZNF423	79	57	0.722	<1.00E-04	8	0.303	YES
PHOX2B	60	42	0.700	<1.00E-04	8	0.275	YES
KLF7	133	92	0.692	<1.00E-04	7	0.313	NO
GLI3	217	150	0.691	<1.00E-04	7	0.325	NO
MEIS1	113	77	0.681	<1.00E-04	8	0.301	YES
KLF2	15	10	0.667	<1.00E-04	8	0.261	YES
TSHZ3	80	52	0.650	<1.00E-04	8	0.302	YES
NKX2-3	75	47	0.627	<1.00E-04	8	0.290	NO
BNC2	252	148	0.587	<1.00E-04	7	0.334	NO
PITX2	26	15	0.577	<1.00E-04	9	0.244	YES
PRDM8	70	40	0.571	<1.00E-04	7	0.304	YES
NR2F2	141	78	0.553	<1.00E-04	7	0.334	YES
TSC22D3	29	16	0.552	<1.00E-04	7	0.282	NO
PBX3	186	102	0.548	<1.00E-04	7	0.337	YES
ZEB1	264	139	0.527	<1.00E-04	7	0.343	YES
FOXF1	62	31	0.500	<1.00E-04	7	0.312	YES
ZNF532	110	52	0.473	<1.00E-04	7	0.326	YES
CAMTA1	68	30	0.441	<1.00E-04	7	0.318	YES
JAZF1	172	74	0.430	<1.00E-04	7	0.334	NO
AFF3	31	11	0.355	<1.00E-04	7	0.295	YES
NFIC	131	43	0.328	<1.00E-04	7	0.339	YES
ZEB2	74	24	0.324	<1.00E-04	7	0.324	YES
TCF4	408	129	0.316	<1.00E-04	6	0.371	YES
BCL6	36	11	0.306	<1.00E-04	7	0.309	NO
NR1H4	77	22	0.286	<1.00E-04	8	0.282	YES
MAFB	125	23	0.184	<1.00E-04	6	0.313	YES
HOXB13	93	14	0.151	<1.00E-04	8	0.264	NO
ATF3	4	3	0.750	0.0003	10	0.184	NO
ZBTB20	149	13	0.087	0.0183982	7	0.356	YES
THRB	12	2	0.167	0.0986901	8	0.259	YES
HOXB6	2	0	0.000	1	8	0.227	YES
IFI16	5	0	0.000	1	7	0.247	YES
NEUROD1	12	0	0.000	1	8	0.236	NO

Genes from the AP-1 complex (*Fosb* and *Jun*) ranked first in the TF list. The AP-1 subunits *Fos*, *Junb*, *Mafb* and *Atf3* also appeared in the list. Previous GSEA analysis also had revealed as most significant motive “*Genes with promoter regions [-2 kb,2 kb] around transcription start site containing the motif TGACTCANNSKN which matches annotation for JUN”* (p-value = 0.002, and FDR q-value = 0.015, Additional file 4: Figure S4). A high correlation existed between the expression of *Jun* and *Fos* AP-1 subunits in adjacent mucosa but not in healthy mucosa (Spearman’s correlation 0.67 and 0.23 respectively; Figure 4B). Interestingly, these TF belonged to the “tumor-like” genes pattern (Figure 4C). *Fos*, *Jun*, *Fosb* and *Junb* did not appear in healthy mucosa transcriptional network highlighting their idiosyncratic role in adjacent mucosa. The family of transcription factors *NR4A1*, *NR4A2* and *NR4A3* also ranked in top positions. Other TF such as *GLI3*, *BCN2*, *EBF1* and *ZEB1* were also significant because of their high rank in the network and large number of DEG targets.

### Deciphering a crosstalk between adjacent mucosa and tumor through a protein-protein interaction network

Changes in adjacent mucosa not detected in healthy mucosa might be a direct response in front of tumor stimulus based on a physical crosstalk between the cells (Figure 1). This molecular communication could be through the direct interaction between secreted proteins and their corresponding membrane receptors. The following strategy was applied to identify interactions compatible with this hypothesis: 1) Search for over-expressed genes in tumors compared to healthy mucosa in addition to previous DEG. 2) Identify those that code for secreted proteins and membrane receptors. 3) Construct a protein interaction network with the selected genes. 4) Identify interaction pairs that reflect cellular communication in both directions: from tumor to adjacent (efferent pathway) and vice versa (afferent pathway).

**Figure 5:**
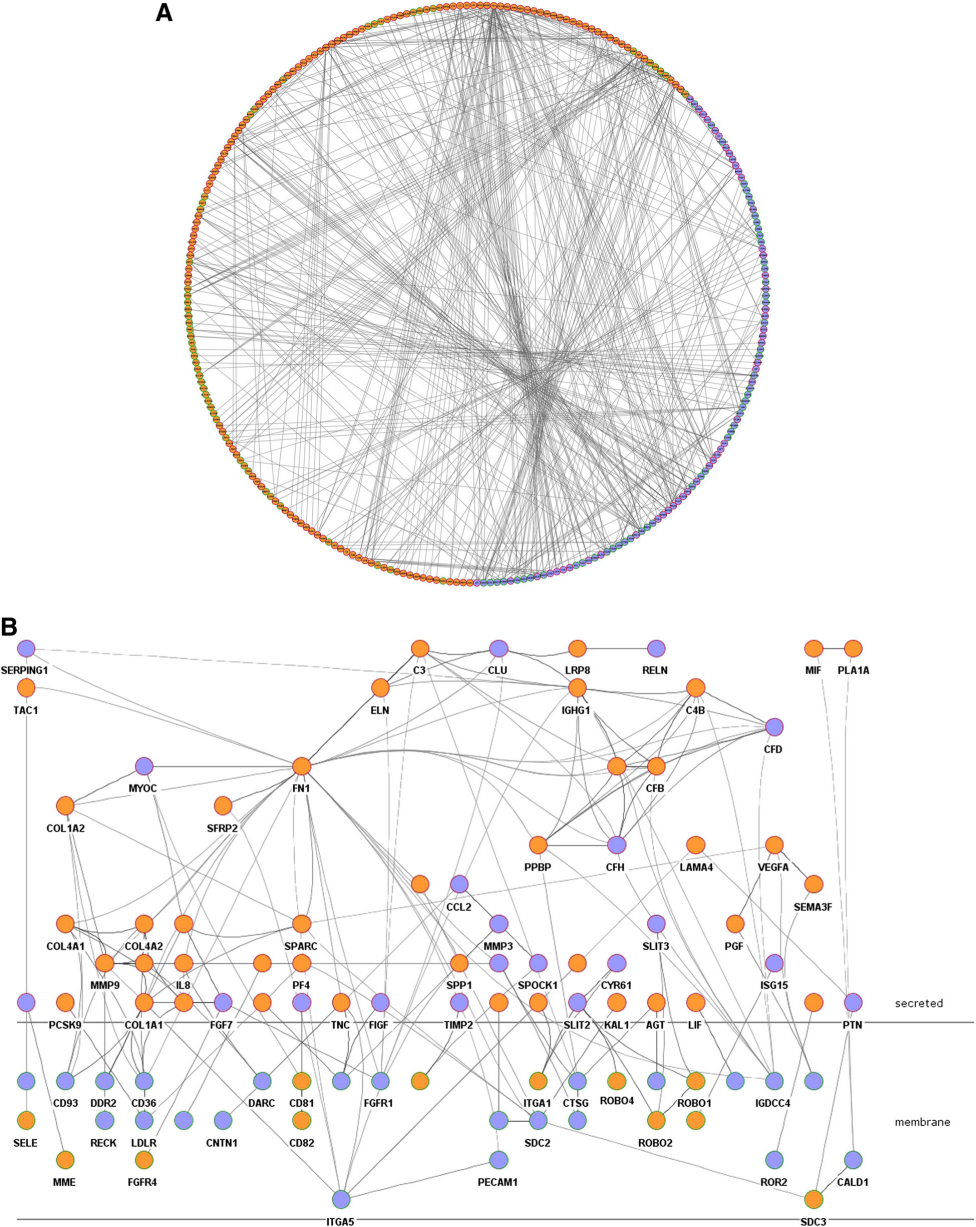
**Crosstalk pathway. A**. Circular layout of protein-protein interaction network representing interactions (lines) between over-expressed genes in adjacent mucosa (purple) and in tumor (orange). Nodes with a green border symbolize membrane proteins whereas red were used to represent secreted proteins **B**. Abstraction of the network in which only crosstalk interactions were drawn, using Cerebral view from Cytoscape.

From the 788 over-expressed genes in adjacent mucosa vs. healthy mucosa, 324 (41%) corresponded to secreted (n = 111) or membrane (n = 213) genes. In addition, 442 genes (250 secreted and 192 membrane) over-expressed in tumors were included in the analysis. A level 0 (only direct interactions) protein-protein interaction network was retrieved using the 766 up-regulated secreted/membrane genes in adjacent and tumor samples as input. The resulting network included 291 nodes connected by 596 interactions, the majority of them integrated in a giant component (Figure 5A). A functional analysis of this network revealed *cell adhesion*, *response to external stimulus*, *response to wounding*, and *anatomical structure development* as the most statistically significant functions (Additional file 7: Table S6).

**Figure 6:**
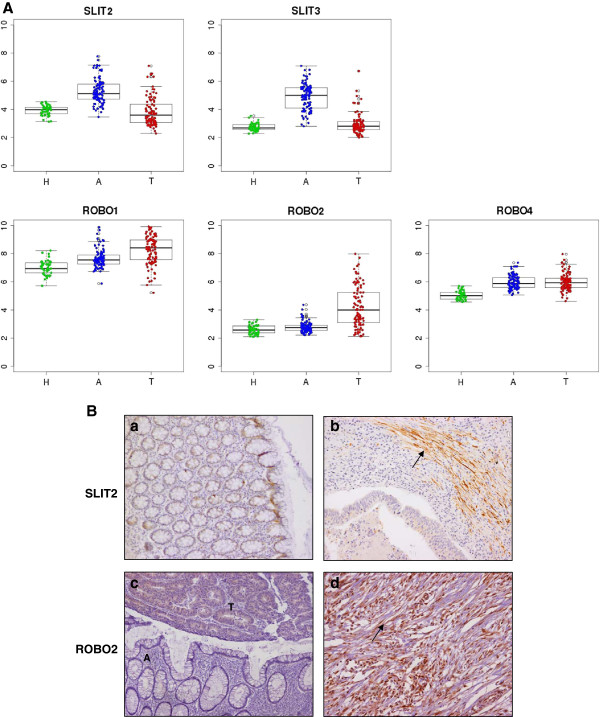
**Slit2 and Robo2 expression. A**. Microarray gene expression for Slit and Robo family of genes. Tumor samples (T) are colored in red, adjacent samples (A) in blue and healthy mucosa (H) in green. **B**. Immunohystochemical staining of Slit2 corresponding to normal epithelial cells from an adjacent mucosa from a cancer-affected patient **(a)**. However, Slit2 antibody stained basically carcinoma-associated fibroblasts and was nearly absent in tumor cells **(b)**. For Robo2, the staining clearly shows how this protein is restricted to tumor tissue, and depending on the patient staining only tumor cells **(c)** or both tumor cells and carcinoma-associated fibroblasts **(d)**. In staining c, tumor and adjacent tissue are marked as T and A. Carcinoma-associated fibroblast are marked with an arrow in b and d photographs.

A curated analysis of the network revealed 84 crosstalk interactions (Table 2), 61 of them efferent (tumor secreted proteins linked to a receptor in adjacent mucosa tissue), and 23 afferent (adjacent mucosa secreted proteins linked to a receptor in tumor). Figure 5B shows an abstraction of the original network restricted to crosstalk interactions. It is remarkable that 6 out of 23 afferent interactions (26%) included members of the Slit family of secreted proteins, which emerged as relevant players in tumor crosstalk determining the adjacent mucosa response. In the network, Slit2 and Slit3 were redundantly activating Robo1, Robo2, Robo4 and ITGA1 receptors in tumor. Slit family followed an adjacent-specific pattern of expression (see Figure 6A). Due to its importance, and as a proof of concept of the overall strategy of gene selection, inmunohistochemical staining was done to asses the protein expression of Slit2 and the receptor Robo2. Slit2 was expressed in adjacent epithelial cells and also in stromal cancer cells. Robo2 was expressed in both epithelial and stromal cells in cancer tissue but not in adjacent tissue (Figure 6B). Other interesting afferent crosstalk pairs involved LRP8 receptor in tumor activated by a double stimulus of RELN and CLU proteins secreted by adjacent mucosa, and VIP, an intestinal peptide that causes vasodilatation, linked to MME receptor in tumor cells.

**Table 2:** Afferent and efferent pairs in the crosstalk network

**AFFERENT PATHWAYS**			
**SECRETED BY ADJACENT MUCOSA**		**LOCATED IN TUMOR MEMBRANE**	
**Gene symbol**	**Function**	**Gene symbol**	**Function**
SLIT2	Cell migration/axon guidance	ITGA1	Integrin-mediated cellular signalling and axon guidance
SLIT2		ROBO2	Cell migration/axon guidance
SLIT2		ROBO1	
SLIT3		ROBO2	
SLIT3		ROBO1	
SLIT2		ROBO4	Cell migration/angiogenesis
FGF7	Growth factor activity	FGFR4	Regulation of cell proliferation, differentiation and migration
PTN	Growth factor with mitogenic activity	SDC3	Organization of cell shape
TIMP2	Metalloendopeptidase inhibitor activity	MMP14	Metalloendopeptidase activit (angiogenesis, cell proliferation…)
MMP3	Regulation of cell migration	MMP14	
SPOCK1	Cell adhesion	MMP14	
ISG15	Interferon-mediated signaling pathway	NEDD4	Virus-host interaction
VIP	Intestinal peptide that causes vasodilation	MME	Cellular response to cytokine stimulus
MYOC	Anatomical structure morphogenesis	CD81	Cell proliferation
RELN	Neuron migration	LRP8	Cytokine-mediated signalling pathway
CLU	Platelet and complement activation	LRP8	
SERPING1	Innate immunity	SELE	Inflammatory response
CYR61	Cell adhesion/chemotaxis	ITGA1	integrin-mediated signaling pathway
FIGF	Angiogenesis	ITGA9	Integrin-mediated signaling pathway, cell adhesion
HBEGF	Growth factor activity	CD82	Metastasis suppressor gene
CXCL12	Immune response	CXCR4	Inflammatory response
CFH	Innate immunity	IGDCC4	Inmunoglobulin
CFD	Innate immunity	IGDCC4	
**EFFERENT PATHWAYS**			
**SECRETED BY TUMOR**		**LOCATED IN ADJACENT MUCOSA MEMBRANE**	
**Gene symbol**	**Function**	**Gene symbol**	**Function**
IL8	Inflammatory response	DARC	Inflammatory response
MIF	Inflammatory response	CALD1	Cellular component movement
PLA1A	Lipid catabolic process	CALD1	
PF4	Immune response and cytokine-mediated signaling pathway	LDLR	Lipid transport and metabolism
WNT5A	In the presence of ROR2, inhibits the canonical Wnt pathway	ROR2	Wnt receptor signaling pathway
REG3A	Inflammatory response	SDC2	Wound healing/carbohydrate metabolic process
SPARC	Regulation of cell proliferation	SDC2	
KAL1	Extracellular matrix structural constituent	SDC2	
PF4	Immune response and cytokine-mediated signaling pathway	SDC2	
FN1	Extracellular matrix structural constituent involved in multiple cellular functions	SDC2	
FN1		ITGA5	Angiogenesis/cell adhesion/wound healing
FN1		IGDCC4	Inmunoglobulin
FN1		CD36	Antigen processing and presentation, lipid storage, cell adhesion
FN1		PECAM1	Cell adhesion, signal transduction
TNC	Guidance of migrating neurons	CNTN1	Notch signaling pathway, cell adhesion
TNC		ITGA5	Angiogenesis/cell adhesion/wound healing
TNC		ITGA9	Integrin-mediated signaling pathway, cell adhesion
COL18A1	Inhibits endothelial cell proliferation and angiogenesis	ITGA5	Angiogenesis/cell adhesion/wound healing
SFRP2	Wnt receptor signaling pathway	ITGA5	
SPP1	Cell adhesion, response to vitamin D	ITGA5	
SPP1		ITGA9	Integrin-mediated signaling pathway, cell adhesion
AGT	Renin-angiotensin system	AGTR1	Inflammatory response, Rho protein signal transduction, Renin-angiotensin system
AGT		CTSG	Immune response
APOC2	Lipid metabolism	IGDCC4	Inmunoglobulin
PPBP	Chemotaxis and inmune response	IGDCC4	
PPBP		CTSG	Immune response
TAC1	Peptide which excite neurons, and are potent vasodilators	TACR2	Response to stress
CXCL5	Chemotaxis and inmune response	DARC	Inflammatory response
CCL2	Chemotaxis and inflammatory response	DARC	
C4A	Inflammatory response	IGDCC4	Inmunoglobulin
IGHG1	Innate inmune response	IGDCC4	
PCSK9	Cellular response to starvation/cholesterol metabolic proces	LDLR	Lipid transport and metabolism
MMP9	Proteolysis	ITGA5	Angiogenesis/cell adhesion/wound healing
MMP9		RECK	Blood vessel maturation
VEGFA	Growth factor active in angiogenesis	NRP2	Angiogenesis
PGF	Growth factor active in angiogenesis	NRP2	
ADAM12	Epidermal growth factor receptor signaling pathway	ITGA9	Integrin-mediated signaling pathway, cell adhesion
COL4A1	Angiogenesis	CD93	Macrophage activation, cell-cell adhesion
COL4A1		CD36	Antigen processing and presentation, lipid storage, cell adhesion
COL1A1	Positive regulation of cell migration/positive regulation of epithelial to mesenchymal transition	DDR2	Cell adhesion, ossification
COL1A1		ITGA5	Angiogenesis/cell adhesion/wound healing
COL1A1		CD93	Macrophage activation, cell-cell adhesion
COL1A1		CD36	Antigen processing and presentation, lipid storage, cell adhesion
COL1A2	Transforming growth factor beta receptor signaling pathway/platelet activation/leukocyte migration	CD93	Macrophage activation, cell-cell adhesion
COL1A2		CD36	Antigen processing and presentation, lipid storage, cell adhesion
COL3A1	Integrin-mediated signaling pathway/blood vessel develpment	DDR2	Cell adhesion, ossification
COL6A1	Axon guidance/cell adhesion	CD36	Antigen processing and presentation, lipid storage, cell adhesion
COL6A3	Axon guidance/cell adhesion	ITGA5	Angiogenesis/cell adhesion/wound healing
COL4A2	Cellular response to transforming growth factor beta stimulus/axon guidance/angiogenesis	CD93	Macrophage activation, cell-cell adhesion
COL4A2		CD36	Antigen processing and presentation, lipid storage, cell adhesion
LAMA4	Cell adhesion	ITGA5	Angiogenesis/cell adhesion/wound healing
CFB	Complement activation	IGDCC4	Inmunoglobulin
SEMA3F	Cell migration	NRP2	Angiogenesis
EFNA3	Cell-cell signalling	EPHA3	Cell adhesion and migration
C3	Complement activation/Fatty acid metabolism	CTSG	Immune response
INHBA	Cell surface receptor signaling pathway	TGFBR3	Negative regulation of transforming growth factor beta receptor signaling pathway
LIF	Growth factor activity	LIFR	Cell proliferation
FN1	Cell adhesion, cell motility, wound healing	FGFR1	Cell proliferation, differentiation and migration
IGHG1	Complement activation	FGFR1	
ELN	Extracellular matrix organization, cell proliferation	FGFR1	
C3	Complement activation	FGFR1	

Efferent interactions were more numerous and included interleukins (IL-8), extracellular-matrix components (Fibronectin, Collagen) or molecules related to invasion like SPARC linked with receptors such as integrins or complement receptors (see Table 2 for specific pairs). Interestingly, the vascular endothelial growth factor receptor NRP2, over-expressed in adjacent mucosa, interacted with a plethora of candidate activating secreted factors from tumors such as VEGFA or SEMA3F. Another interesting finding was the over-expression of LIF in tumor, whose receptor LIFR was over-expressed in adjacent mucosa but not in the tumor. These results were highly indicative of an active crosstalk between cells in the gut microenvironment that triggers an intra-cellular signaling response. The protein-protein interaction network also revealed autocrine signals within tumor or adjacent mucosa. For example, the vascular endothelial growth factor receptor FLT1 was found linked with its ligand VEGFA, both over-expressed in tumor samples.

The bulk tumor includes a mixture of epithelial and active stromal cells. In order to assess which compartment was predominantly expressing the proteins involved in the identified crosstalk interactions, we used the expression data described by Calon et al. [4] who analyzed profiles of each cell population sorted from human CRC. As a result, the vast majority of genes were over-expressed in the stromal compartment (i.e. collagens, interleukins) indicating their active role in the remodeling of the surrounding microenvironment (Additional file 8: Table S7).

To look for hypothetical relationships explaining the communication loop between TF and membrane receptors activated in CRC-adjacent mucosa, a network using as seed proteins AP-1 and membrane receptors was retrieved. Only experimentally-determined interactions were used to construct this level 1 PPIN (including proteins working as bridges between seed proteins that add information to the studied system). As a result, a strong physical interaction between these two cellular components (the extracellular one and the nuclear one) was found. Twenty-one membrane receptors (out of 22) interact with each other through linker proteins to transduce a cellular signal across the extracellular matrix and membrane, finally activating TF belonging to the AP-1 complex (Additional file 4: Figure S5). It is remarkable the close relationship found between ITGA9, ITGA5, CD36, CD93, TGFBR3 and RECK receptors. Also, this analysis revealed a direct path from ROR2 receptor and the AP-1 transcriptional sub-network, being the ligand WNT5A (up-regulated in tumor tissues) the activator of this signal.

## Discussion

There is clear evidence of the relevance of the tumor-microenvironment crosstalk for carcinogenesis [12-15]. Here we describe altered patterns of expression of the adjacent mucosa from colon cancer patients that could be a direct response against the tumor or induced by the tumor. The analysis of transcriptional profiles and the regulatory networks derived from them allowed us identifying the pathways involved in tumor-microenvironment crosstalk.

We can not discard that at least part of the differences found between adjacent and healthy mucosa were explained by the existence of a pre-neoplastic field in the gut. Studies of adjacent mucosa of the head and neck tumors indicate that such fields can expand more than 7 cm in diameter [10]. Nevertheless, a study in CRC by Jothy S. et al. reported a gradient of carcinoembryonic antigen (CEA) expression expanding only 5 cm. from the peritumor area [16]. In our study, adjacent tissue from patients was dissected from the proximal tumor resection margin, with a minimum distance of 10 cm. However, a recent paper by Hawthorn et Mojica suggests that the field effect cancerization could be evident up to 10 cm. from the tumor [17].

Previous studies usually have compared paired tumor and adjacent mucosa tissues, which can result in misleading interpretations. We have used a large sample of healthy mucosa as reference for gene expression comparisons and have identified a large number of DEG that can be grouped into three altered patterns: “tumor-like”, “trend”, and “adjacent-specific”. Our conclusion is that adjacent normal mucosa is not so normal. In fact, studies that only compare tumor and adjacent mucosa may miss good cancer biomarkers candidates, because many genes are deregulated in adjacent mucosa mimicking the tumor expression.

The predominant functions of DEG are mainly related to response to stimulus, extracellular matrix (ECM) remodeling, organ morphogenesis, and cell adhesion. Remodeling of the ECM network though controlled proteolysis regulates tissue tension, generate pathways for migration, and release ECM protein fragments to direct normal developmental processes such as branching morphogenesis [8]. Collagens are major components of the ECM of which basement membrane type IV and interstitial matrix type I are the most prevalent. Abnormal expression, proteolysis and structure of these collagens influence cellular functions to elicit multiple effects on tumors, including proliferation, initiation, invasion, metastasis, and therapy response [18]. It has been described that integrins that connect various cell types play a vital role in the survival of a growing tumor mass by orchestrating signaling pathways activated through cell-cell and cell-matrix interactions [6]. In our system, integrins ITGA5 and ITGA9 emerged as active signal transducers, occupying central positions in the cellular networks. This result suggests that integrins are not only vital proteins in tumor cells but also in normal-adjacent cells. Moreover, our results indicate that proteins implicated in the described crosstalk are predominantly over-expressed by the tumor stroma. This result underscores the important role of this compartment in CRC carcinogenesis.

One important finding is that DEG are enriched in transcription factors. This indicates the existence of a transcriptional program driving the altered expression pattern observed in adjacent mucosa. A loop including members of the AP-1 family of transcription factors emerged as the most significant one in the analysis. Interestingly, these TF are over-expressed in both adjacent mucosa and tumor tissue. AP-1 members homo or hetero dimerize to assemble the activator protein 1 (AP-1). AP-1 transcription factor acts synergistically with SMAD3/SMAD4 component and is implicated in the regulation of a variety of cellular processes including proliferation and survival, differentiation, growth, apoptosis, cell migration, and inflammation [19,20]. Topologically, these nodes have a low centrality but a high eccentricity in the transcriptional network. This result can be a little controversial since it is widely accepted that the more centered a node is the more important their functional role in the studied system [21]. However, a recent publication postulates that nodes with high eccentricity could be quickly activated by external factors [22]. This observation could explain the radial position of AP-1 members Jun, Fos, FosB and JunB into the transcriptional network as important fast effectors mediating response against the tumor.

We hypothesized that cytokines and other signaling proteins secreted by the tumor activate membrane receptors of adjacent mucosa cells that initiate this transcription factor activity. Tumor-secreted growth factors act as paracrine agents distorting the normal tissue homeostasis. In turn, tumors are both maintained or attacked by signals from the surrounding microenvironment inducing stromal reaction, angiogenesis and inflammatory responses. To gain insight into the molecular mechanisms underlying this phenomenon, a bi-tissue PPIN analysis strategy was performed to extract patterns of receptor activation in both directions from adjacent mucosa to tumor and vice-versa. Robo genes appeared as the most recurrently receptors activated in tumor membrane by Slit family of proteins. Slits have been implicated in regulating a variety of life activities, such as axon guidance, neuronal migration, neuronal morphological differentiation, tumor metastasis, angiogenesis and heart morphogenesis [23]. Several studies have demonstrated dual roles for Slit and Robo in cancer, acting as both oncogenes and tumor suppressors [24]. This bi-functionality is also observed in their roles as axon guidance cues in the developing nervous system, where they both attract and repel neuronal migration [25]. In CRC, Slit2 up-regulation has been reported as beneficial for the overall survival of patients [26]. Slit is under-expressed in patients with metastatic colorectal cancer and their over-expression in cells resulted in an inhibition of cell migration through AKT-GSK3β signaling pathway. In our data, no significant association between Slit2 or Slit3 level of expression and prognosis was found.

CLU-RELN-LRP8 was other afferent axis to consider for further analysis. CLU codifies the protein Clusterin that has been described as both tumor suppressor and pro-survival factor in colon cancer depending on the intra- and extracellular microenvironment crosstalk [27]. In fact, it has been reported that Clusterin is a protein that shares the intracellular information with the microenvironment and it also experiences a systemic diffusion, acting as a factor that synergistically interacts with their surrounding microenvironment [28]. Moreover, it has been proposed as a diagnostic biomarker in colon cancer [29]. The other CRC-mucosa-secreted protein activating LRP8 receptor in tumor is Reelin (*RELN*), a glycoprotein that plays an important role in neuronal migration through the activation of lipoproteins receptors such as LRP8 [30]. Also, Reelin has been proposed as a pro-metastatic factor due to their role in cancer cell migration through TGF-β pathway activation [31].

Efferent pathways were also of interest. LIF is a member of the IL6 family of cytokines that displays pleiotropic effects on various cell types and organs [32,33]. In our system, its receptor LIFR was expressed in the colonic epithelium. It has been reported that LIF stimulates the Jak/STAT pathway to produce nitric oxide (NO) [34,35]. Based on this, we hypothesize that, in our model, tumor LIF activates Jak-STAT pathway in normal epithelial cells through LIFR receptor leading to NO release and the subsequent creation of a pro-inflammatory environment. Moreover, in our model, Angiotensinogen (AGT) was produced by the tumor and their receptor (AGTR1) was located in membrane from adjacent tissue. Since Angiotensinogen is the precursor form of the active peptide Angiotensin, the pair AGT-AGTR1 makes up the renin-angiotensin system (RAS), usually associated with cardiovascular homeostasis but recently associated with tumor growth [36]. RAS could play a synergistic effect with LIF inducing NO production, leading to inflammation, macrophage infiltration and tumor-induced fibrosis. In addition to their pro-inflammatory role, it has been reported that NO can activate notch-signaling pathway leading to the induction of tumors [37].

Conceptually, an active sub-network includes differentially expressed and connected proteins in a given phenotype. Here we have described a sub-network including membrane receptors over-expressed in normal adjacent tissue acting together in cell-adhesion and with functions on cell surface signal transduction that finally activate the AP-1 transcription factor. ROR2 has emerged as an important link in the crossroad between cell surface entering signal and Fos/Jun transcriptional role as previously described [38]. ROR2 is tyrosine-kinase receptor that plays an important role in developmental morphogenesis [39] and in our network it was activated by the tumor-secreted WNT5A, a WNT pathway signaling mediator.

We do not exclude the possibility that genes having a pivotal role in crosstalk between adjacent and tumor tissue also have a direct relationship with prognosis. In our data, expression of Fos and Jun were found to be protective when over-expressed in adjacent but not in tumor tissue (log-rank p-value = 0.042). Further studies are needed to experimentally corroborate this hypothesis and to test the utility of these transcription factors as prognosis biomarkers. Nevertheless, a complex equilibrium between positively pro-survival and pro-apoptotic signals given by the microenvironment ultimately influences the tumor growth and their plasticity. This could be one of the reasons why prognosis signatures that only take into account tumor but not adjacent tissue expression fail to accurate predict patients’ outcome [40].

The study has some methodological and technical limitations. Though we obtained adjacent mucosa from the farthest resection margin and usually required at least 10 cm, it is possible that some of the variability observed among adjacent mucosa might be related to the distance to the tumor that we cannot analyze. Also, despite a careful dissection of tumor blocks before RNA extraction was done, a normal adjacent tissue infiltration can exist in some tumor samples. Regarding analytical methods, the network analysis only considered well-annotated genes. Some TFs were excluded from the transcriptional network analysis due to their low variability in our data. For these reasons, some genes with a putative role in colon tissue remodeling could have been missed. In fact, we did not find *TGF-β*, proposed as an important microenvironment modifier [4] because its probeset had very low expression level in our microarray. Finally, our study only included colon specimens, which could raise a concern about generalizability of the results. However, we have previously analyzed that the expression levels are very similar in colon and rectal tumors [41] and this has been confirmed in the TCGA study [42].

## Conclusions

In conclusion, gene expression in cells comprising normal adjacent tissue in CRC patients is not so normal and this could have important implications in colorectal cancer prognosis and progression. A systems-level approach has been useful to gain insight into the molecular mechanisms by which adjacent mucosa activates a transcriptomic program in response to cytokines and other signaling proteins secreted by the tumor. We hypothesize that a crosstalk exists, not only between different cell communities within the tumor bulk, but also between colorectal tumor cells and adjacent mucosa, which reacts against the tumor like against a wound. Tumor-secreted growth factors act as paracrine agents distorting the normal tissue homeostasis. In turn, tumors are both maintained and/or attacked by signals from the surrounding microenvironment inducing stromal reaction, angiogenesis and inflammatory responses. Disrupting this intricate molecular network of cell-cell communication and signal transduction could be a therapeutic target in CRC patients.

## Methods

### Patients and samples

A set of 98 paired adjacent normal and tumor tissues from CRC patients and 50 colon mucosa from healthy donors (246 samples in total) were included in this work. Patients were selected to form a homogeneous clinical group of stage II, microsatellite stable (MSS) colorectal tumors. All had been treated with radical surgery, had not received adjuvant therapy and had a minimum follow up of three years. Adjacent normal tissue from patients was dissected from the proximal tumor resection margin with a minimum distance of 10 cm. Healthy donors were invited to participate in this study when they underwent a colonoscopy indicated for screening or symptoms with no evidence of lesions in the colon or rectum (Additional file 9: Table S8). In this paper we use tumor (T), adjacent mucosa (A) and healthy mucosa (H) to designate the different tissue origins for the samples analyzed. All patients were recruited at the Bellvitge University Hospital (Spain) and the Ethics Committee approved the protocol. Written informed consent from patients and healthy donors was required for inclusion in this study.

### Differential expression analysis

RNA extracted from each sample was hybridized in Affymetrix chips Human Genome U219. After a quality control assessment following Affymetrix standards, data was normalized using the RMA algorithm [43]. Both raw and normalized data are available in the NCBI’s Gene Expression Omnibus (GEO) database [44] through accession number GSE44076.

Prior to the identification of differentially expressed genes, a filter was applied to remove low variability probes (n = 15,533), which mostly corresponded to non-hybridized and saturated measures. The remaining 33,853 probes showed a standard deviation greater than 0.3 and were considered for further analysis. A t-test was used to identify differences in gene expression between apparently normal adjacent mucosa from CRC patients (A) and mucosa from healthy donors (H). A probe was considered differentially expressed when it was significant at 1% FDR (q-value method) and showed an absolute log2 mean difference higher than 1 (double expression). The same criteria were applied to identify differentially expressed genes between tumor (T) and healthy mucosa (H).

To attempt a validation of the differentially expressed genes, the same methods were applied to compare samples of healthy colonic mucosa (n = 13) and adjacent mucosa (n = 24) extracted from public datasets GSE38713 [45] and GSE23878 [46].

### Functional analysis

Pathway enrichment analysis was performed using two methods. First, *Sigora R* package [47] was used, which focuses on genes or gene-pairs that are (as a combination) specific to a single pathway. *Sigora* contains pre-computed data for human pathways in the KEGG [48], BIOCARTA [49], NCI [50], INOH [51] and REACTOME [52] repositories. Second, the gene set enrichment analysis (GSEA) algorithm was also applied, which uses the ranking of differences to identify pathways from a large list of pre-specified sets [53].

### Analysis of transcription factors

Transcriptional networks attempt to translate gene expression correlations into transcriptional relationships to reconstruct regulatory loops between transcription factors and their target genes. Transcriptional regulation networks had been previously inferred using the ARACNe algorithm [54], which identifies direct regulatory associations between transcription factors and targets from mutual information measures of co-expression. The associations, represented as a transcriptional network, were used to identify and characterize transcription factors de-regulated in adjacent mucosa from patients when compared to healthy mucosa. Deregulated transcription factors were ranked using a score that took into account both topological parameters of the network and the node expression values. This score divided the number of deregulated nodes linked to each transcription factor by the total number of nodes linked to the transcription factor. To assess statistical significance, a p-value was calculated by re-sampling 1000 times random lists of genes. For each transcription factor, the Network Analyzer module [55] from Cytoscape [56] was used to extract the topological parameters closeness centrality and eccentricity. Only those genes annotated as transcription factor based on experimental data were used in this analysis, whereas those annotated “in silico” were not considered [57,58].

### Protein-protein interaction network construction and analysis

Protein interaction data can be represented as networks were nodes represent proteins and edges represent physical interactions between them. BIANA software (Biological Interactions and Network Analysis) was used to retrieve such networks [59]. BIANA builds networks by selecting interacting partners for an initial set of seed proteins (i.e., the relevant proteins), combining experimentally-determined data from DIP [60], MIPS [61], HPRD [62], BIND [63] and the human interactions from two high-throughput experiments [64,65]. The integration of multiple sources of interaction data into a single repository allows working with an extensive set of interactions. For our analysis, only human and experimentally-determined interactions were taken into account. Cytoscape software and its plug-ins were used to analyze and visualize the networks.

### Cellular classification of tumor proteins implicated in the crosstalk

Proteins were classified as “epithelial” or “stromal” on the basis of their gene level of expression in specific cellular subtypes. For this classification, normalized data from the public dataset GSE39396 was used, which included 24 samples corresponding to different human CRC cell populations: epithelial, endothelial, fibroblasts and leukocytes [4].

### Immunohistochemistry

Slices of paraffin-embedded tissue (4 μm thick) from 5 pairs of matched samples adjacent-mucosa tumor tissue were used. For antigen retrieval, the slides were boiled after deparaffinization in a pressure cooker for 10 minutes in citrated buffer (8.2 mM tri-sodium citrate and 1.98 mM citric acid, pH6) for Robo2 detection and in EDTA buffer (1 mM EDTA, 0.05% Tween-20, pH8) for Slit2 detection. Endogenous peroxidase was blocked with 3% H_2_O_2_ during 20 minutes. After blocking during 30 minutes with 1/5 dilution of goat serum, primary antibodies were incubated overnight at 4°C. Primary antibodies were rabbit polyclonal against Slit2 (Abcam, ab111128) and rabbit polyclonal against Robo2 (Prestige Antibodies, HPA013371), diluted both 1:100 in antibody diluent (Dako, Copenhagen, Denmark). Reaction was visualized using EnVision anti-rabbit antibody system, and developed using DAB-Plus Kit (Dako). Slides were counterstained with Harry’s modified haematoxylin. As negative control we used EnVision anti-rabbit antibody system and displayed no reactivity against any antigen.

## Abbreviations

BIANA: Biological interactions and network analysis; CRC: Colorectal cancer; DEG: Differentially expressed genes; ECM: Extracellular matrix; EMT: Epithelial to mesenchymal transition; DETF: Differentially expressed transcription factors; MSS: Microsatellite stable; PPIN: Protein-protein interaction network; TF: Transcription factor.

## Competing interest

The authors have declared that no competing interests exist.

## Authors’ contributions

RSP and VM designed the study, conceived the experiments and wrote the article. RSP and AB carried out the experiments. DC performed transcriptional networks. DGM performed inmunohistochemistry and helped to draft the manuscript. XSo, MCB, LPB, and EG analyzed data. CS, JO, XSa, FRM and RS provided samples and clinical data. All authors critically reviewed and had final approval of the article.

## Supplementary Material

Additional file 1: Table S1 List of DEG between adjacent and healthy mucosa. Click here for file

Additional file 2: Table S2 Sigora functional analysis results. Click here for file

Additional file 3: Table S3 GSEA functional analysis results. Click here for file

Additional file 4: Figure S1 GSEA representative results. Red and blue bar stands for adjacent and healthy mucosa, respectively. **Figure S2.** Venn diagram shows the intersection between DEG in our patients series and DEG in the validation series, both at FDR 1% and FC >2 (adjacent vs. healthy mucosa). The heatmap on the right shows how DEG extracted from our discovery set are able to correctly classify healthy and adjacent samples in the validation set. Highlighted in black, the group of adjacent samples showing an extreme phenotype. **Figure S3.** Transcriptional regulation networks of adjacent **(A)** and healthy mucosa **(B)** tissues. **Figure S4.** GSEA term “Genes with promoter regions [-2 kb,2 kb] around transcription start site containing the motif TGACTCANNSKN which matches annotation for JUN: jun oncogene”. Red and blue bar stands for adjacent and healthy mucosa, respectively. **Figure S5.** Protein-protein interaction network showing the axis membrane receptors – AP-1 transcription factors, activated in adjacent mucosa. Seed proteins are colored in green (transcription factors) or brown (membrane receptors), and highlighted in grey. Inferred interacting proteins are colored in light purple. Click here for file

Additional file 5: Table S4 Gene expression levels of the 895 DEG between adjacent and mucosa samples, in independent public datasets GSE38713 and GSE23878. Only 825 out of 895 genes were found in the validation serie microarray. Click here for file

Additional file 6: Table S5 List of significant functions stratified by pattern. Click here for file

Additional file 7: Table S6 Crosstalk network functional analysis. Click here for file

Additional file 8: Table S7 Origin of proteins implicated in the crosstalk which are secreted by the tumor or located in tumor membrane. Click here for file

Additional file 9: Table S8 Baseline characteristics of healthy donors and CRC patients. Click here for file
