# Supplementary material for: Aberrant gene expression in mucosa adjacent to tumor reveals a molecular crosstalk in colon cancer
Source: Mol Cancer. 2014 Mar 5;13:46. doi: 10.1186/1476-4598-13-46 (PMC4023701; doi:10.1186/1476-4598-13-46)
Supplement: Additional file 2: Table S2 — Sigora functional analysis results. [file 1476-4598-13-46-S2.doc]

Supplementary Table 2. Pathway’s enrichment analysis between adjacent mucosa and healthy mucosa (Sigora software).

|  | Significant pathways | Adjusted p-value |
| --- | --- | --- |
| **BIOCARTA** | Inhibition of matrix metalloproteinases | 2.82E-18 |
|  | Pertussis toxin-insensitive ccr5 signaling in macrophage | 2.46E-05 |
| **KEGG** | Cell adhesion molecules (CAMs) | 1.24E-157 |
|  | Focal adhesion | 6.77E-106 |
|  | Protein digestion and absorption | 2.73E-104 |
|  | Vascular smooth muscle contraction | 1.32E-97 |
|  | Complement and coagulation cascades | 4.40E-71 |
|  | ECM-receptor interaction | 6.51E-64 |
|  | Regulation of actin cytoskeleton | 4.31E-28 |
|  | Malaria | 9.25E-27 |
|  | MAPK signaling pathway | 3.98E-19 |
|  | TGF-beta signaling pathway | 8.56E-19 |
|  | Leukocyte transendothelial migration | 6.89E-18 |
|  | Pathways in cancer | 3.43E-16 |
|  | Hepatitis C | 1.18E-14 |
|  | Cytokine-cytokine receptor interaction | 1.47E-14 |
|  | Axon guidance | 1.38E-13 |
|  | Tight junction | 1.83E-13 |
|  | Prion diseases | 8.94E-13 |
|  | Gap junction | 5.49E-09 |
|  | Wnt signaling pathway | 1.20E-08 |
|  | Renin-angiotensin system | 2.25E-08 |
|  | Circadian rhythm - mammal | 1.05E-07 |
|  | Calcium signaling pathway | 3.38E-07 |
|  | PPAR signaling pathway | 4.70E-07 |
|  | Phagosome | 1.73E-06 |
|  | Neuroactive ligand-receptor interaction | 2.31E-06 |
|  | RIG-I-like receptor signaling pathway | 2.98E-06 |
| **REACTOME** | Microtubule-dependent trafficking of connexons from Golgi to the plasma membrane | 0 |
|  | BoNT Light Chain Types A, C1, E cleave SNAP-25 | 0 |
|  | COX reactions | 0 |
|  | Fructose catabolism | 0 |
|  | Integrin cell surface interactions | 8.02E-102 |
|  | Smooth Muscle Contraction | 3.70E-84 |
|  | Interferon alpha/beta signaling | 3.28E-49 |
|  | Regulation of IGF Activity by IGFBP | 7.42E-45 |
|  | Transcriptional Regulation of White Adipocyte Differentiation | 1.12E-34 |
|  | Chemokine receptors bind chemokines | 9.98E-33 |
|  | Cell-extracellular matrix interactions | 1.28E-14 |
|  | Interaction between L1 and Ankyrins | 1.52E-06 |
|  | Nectin/Necl trans heterodimerization | 3.37E-06 |
|  | Activation of the AP-1 family of transcription factors | 3.37E-06 |
|  | Hormone-sensitive lipase (HSL)-mediated triacylglycerol hydrolysis | 4.26E-06 |
|  | Tachykinin receptors bind tachykinins | 9.25E-06 |
|  | Alternative complement activation | 1.18E-05 |
|  | cGMP effects | 2.06E-05 |
|  | Nitric oxide stimulates guanylate cyclase | 2.06E-05 |
| **NCI** | Beta1 integrin cell surface interactions | 4.56E-63 |
|  | AP-1 transcription factor network | 3.95E-22 |
|  | Validated transcriptional targets of AP1 family members Fra1 and Fra2 | 6.16E-13 |
|  | FGF signaling pathway | 1.89E-08 |
|  | Beta3 integrin cell surface interactions | 3.89E-08 |
|  | Syndecan-4-mediated signaling events | 1.15E-06 |
| **INOH** | Integrin signaling pathway | 5.14E-120 |
|  | IGF1 signaling pathway | 1.51E-08 |
