# Supplementary material for: Aberrant gene expression in mucosa adjacent to tumor reveals a molecular crosstalk in colon cancer
Source: Mol Cancer. 2014 Mar 5;13:46. doi: 10.1186/1476-4598-13-46 (PMC4023701; doi:10.1186/1476-4598-13-46)
Supplement: Additional file 4: Figure S1 — GSEA representative results. Red and blue bar stands for adjacent and healthy mucosa, respectively. Figure S2. Venn diagram shows the intersection between DEG in our patients series and DEG in the validation series, both at FDR 1% and FC >2 (adjacent vs. healthy mucosa). The heatmap on the right shows how DEG extracted from our discovery set are able to correctly classify healthy and adjacent samples in the validation set. Highlighted in black, the group of adjacent samples showing an extreme phenotype. Figure S3. Transcriptional regulation networks of adjacent (A) and healthy mucosa (B) tissues. Figure S4. GSEA term “Genes with promoter regions [-2 kb,2 kb] around transcription start site containing the motif TGACTCANNSKN which matches annotation for JUN: jun oncogene”. Red and blue bar stands for adjacent and healthy mucosa, respectively. Figure S5. Protein-protein interaction network showing the axis membrane receptors – AP-1 transcription factors, activated in adjacent mucosa. Seed proteins are colored in green (transcription factors) or brown (membrane receptors), and highlighted in grey. Inferred interacting proteins are colored in light purple. [file 1476-4598-13-46-S4.ppt]

## Slide 1
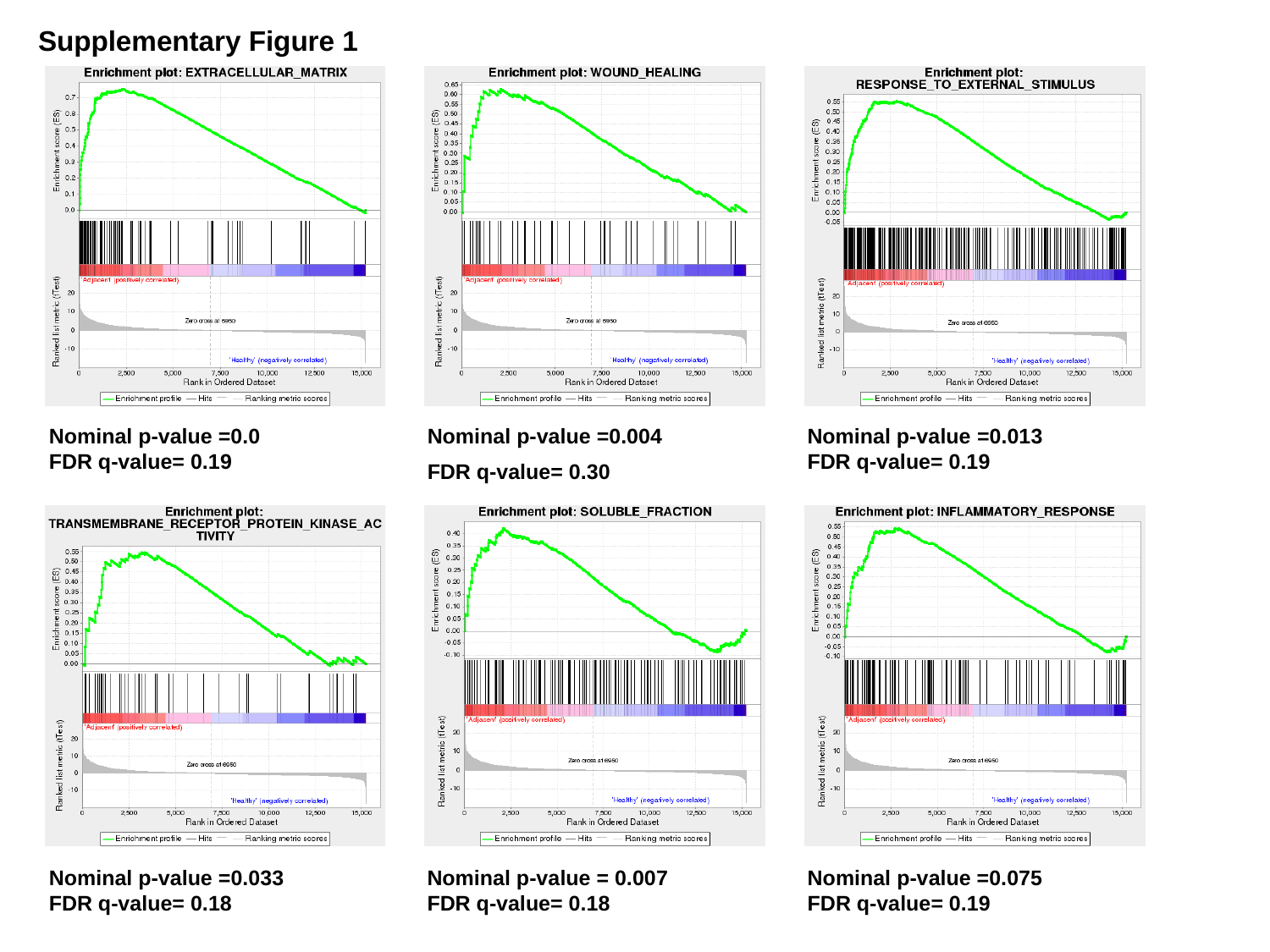

Supplementary Figure 1
Nominal p-value =0.0
FDR q-value= 0.19
Nominal p-value =0.004
FDR q-value= 0.30
Nominal p-value =0.013
FDR q-value= 0.19
Nominal p-value =0.033
FDR q-value= 0.18
Nominal p-value = 0.007
FDR q-value= 0.18
Nominal p-value =0.075
FDR q-value= 0.19

## Slide 2
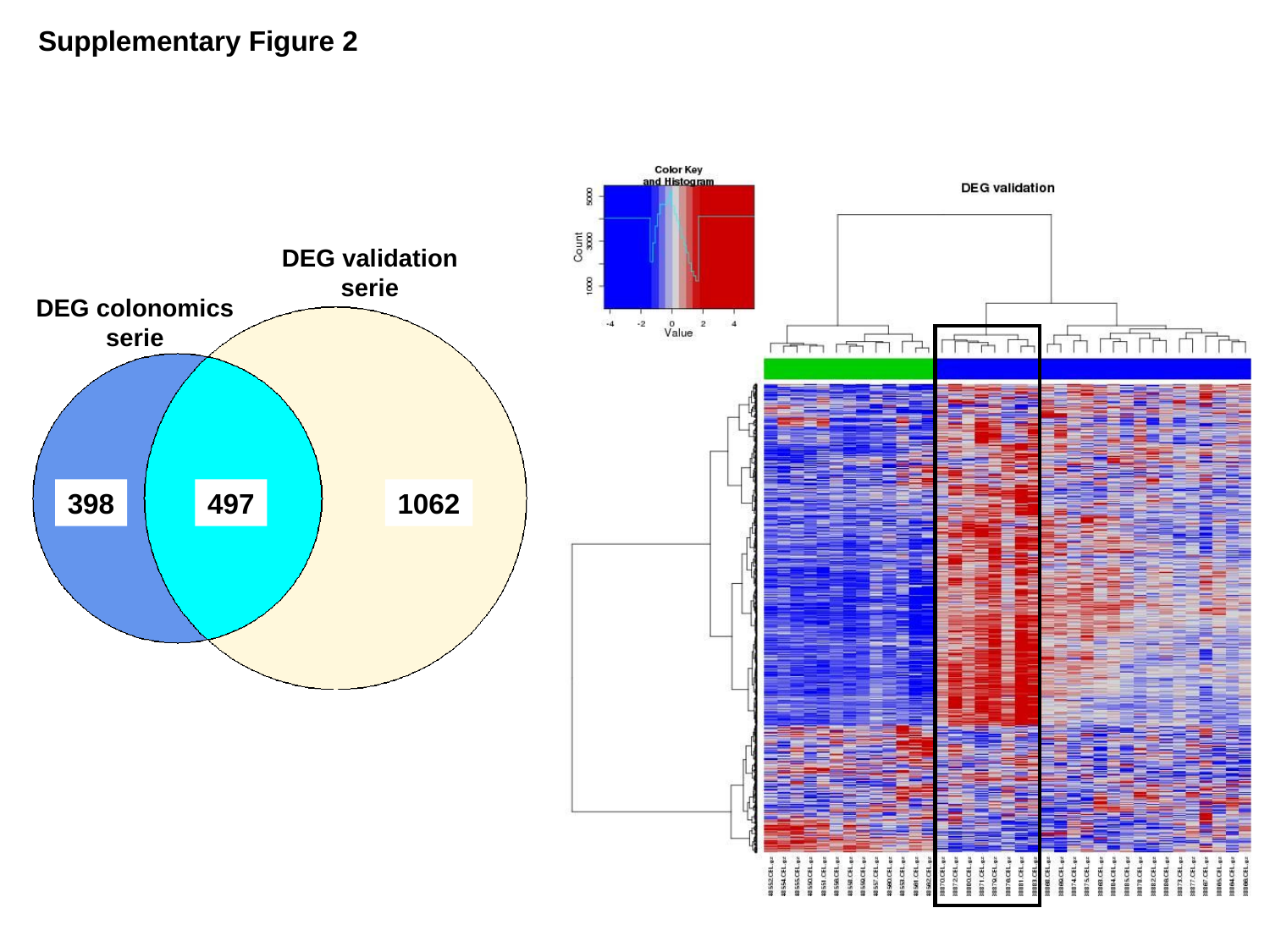

Supplementary Figure 2
DEG validation serie
DEG colonomics serie
398
497
1062

## Slide 3
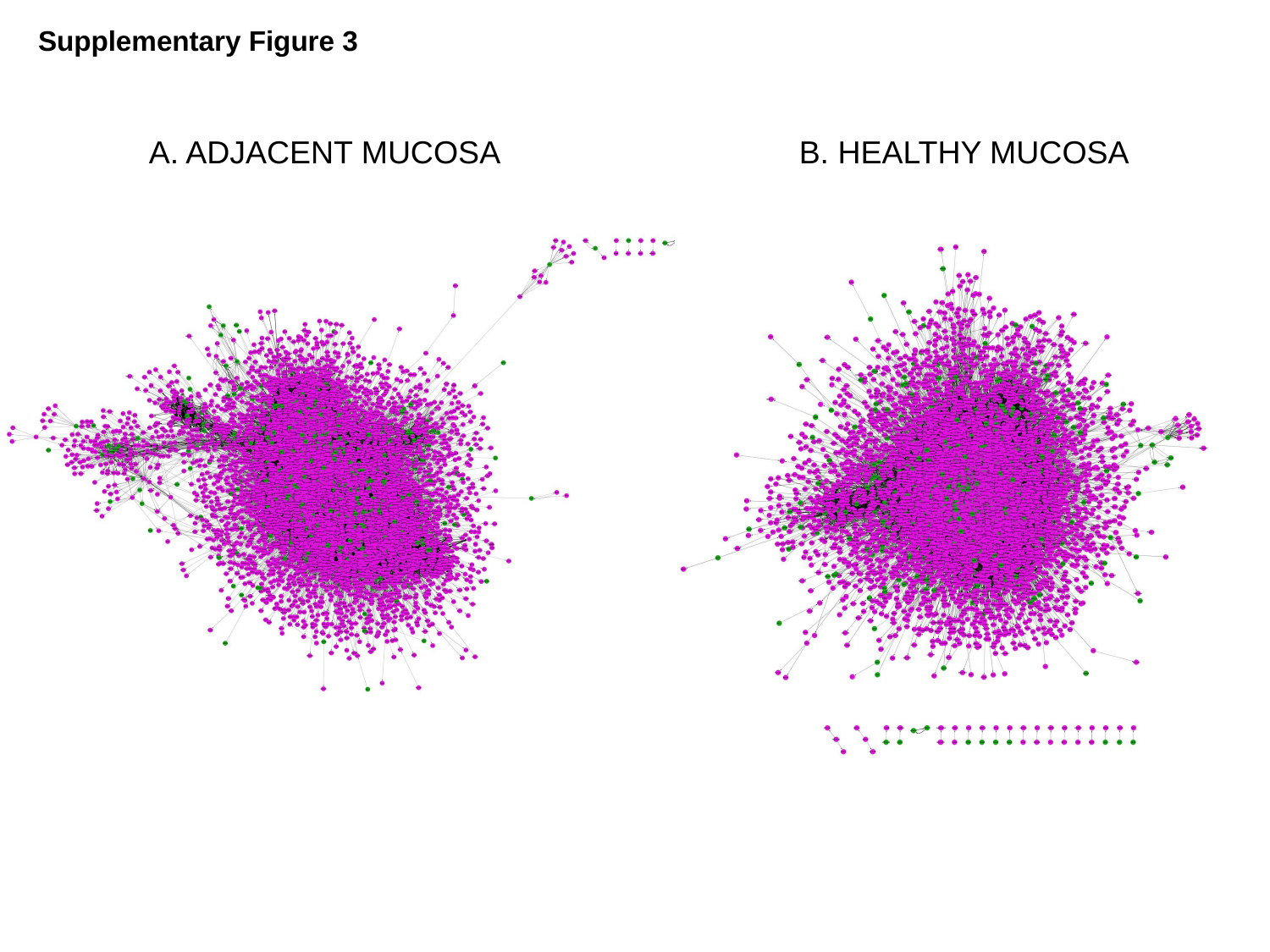

Supplementary Figure 3
A. ADJACENT MUCOSA
B. HEALTHY MUCOSA

## Slide 4
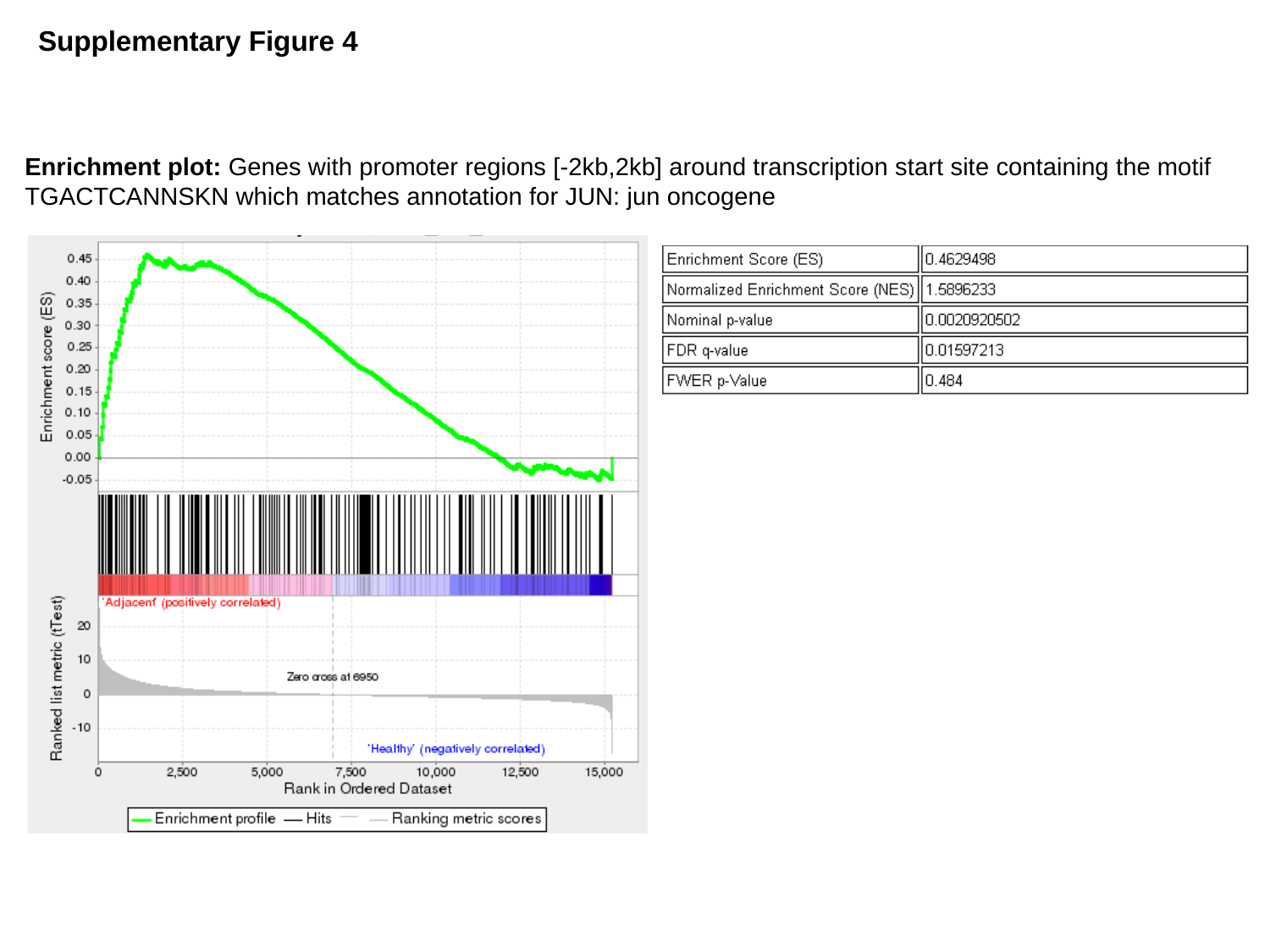

Supplementary Figure 4
Enrichment plot: Genes with promoter regions [-2kb,2kb] around transcription start site containing the motif TGACTCANNSKN which matches annotation for JUN: jun oncogene

## Slide 5
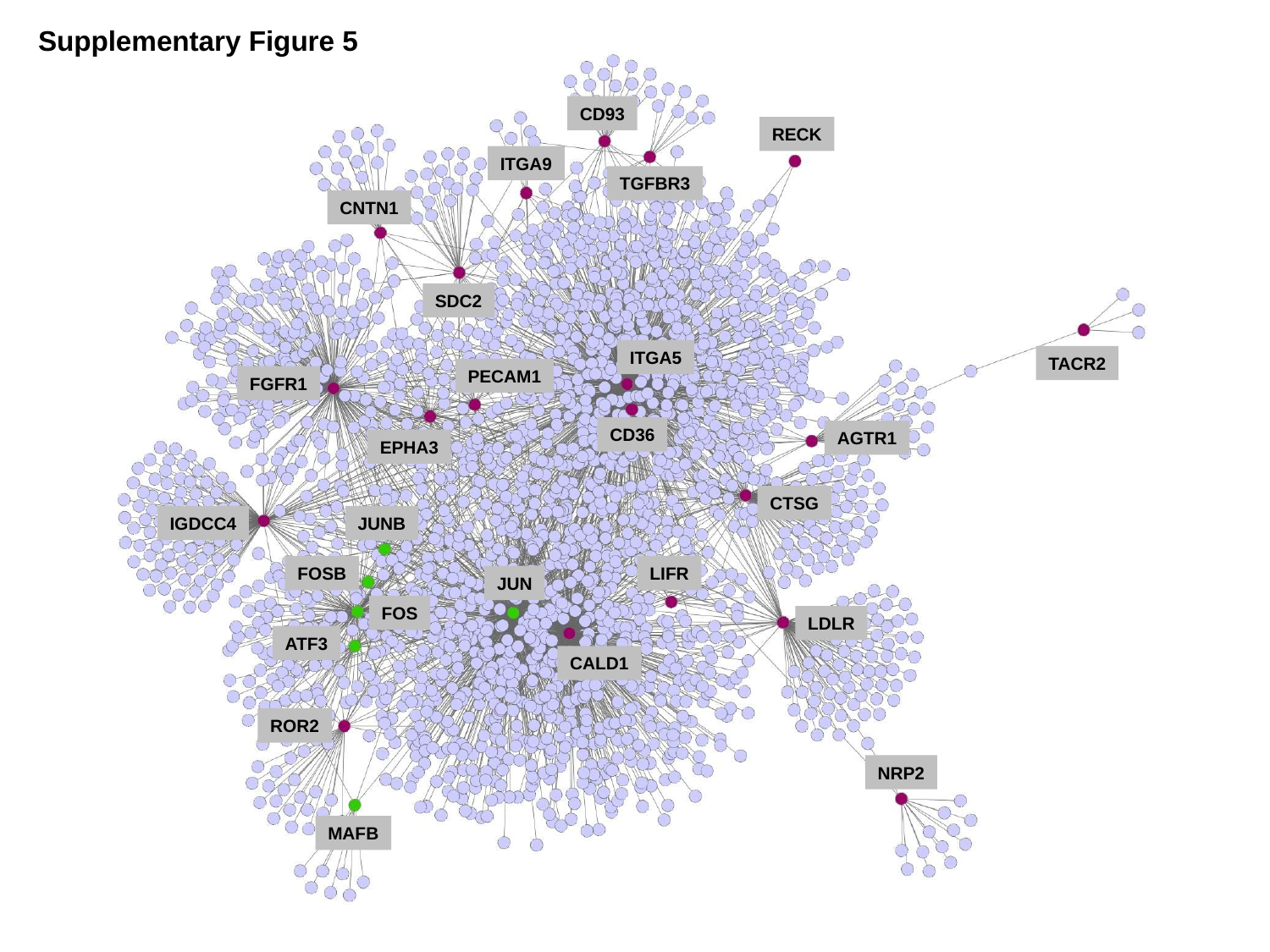

Supplementary Figure 5
CD93
RECK
ITGA9
TGFBR3
CNTN1
SDC2
ITGA5
TACR2
PECAM1
FGFR1
CD36
AGTR1
EPHA3
CTSG
IGDCC4
JUNB
FOSB
LIFR
JUN
FOS
LDLR
ATF3
CALD1
ROR2
NRP2
MAFB
