# Supplementary material for: Aberrant gene expression in mucosa adjacent to tumor reveals a molecular crosstalk in colon cancer
Source: Mol Cancer. 2014 Mar 5;13:46. doi: 10.1186/1476-4598-13-46 (PMC4023701; doi:10.1186/1476-4598-13-46)
Supplement: Additional file 6: Table S5 — List of significant functions stratified by pattern. [file 1476-4598-13-46-S6.doc]

Supplementary Table 5. Pathway’s enrichment analysis within different patterns of gene expression

|  | **pathways in adjacent-specific genes** | **p-value** | **pathways in tumor-like genes** | **p-value** | **pathways in trend genes** | **p-value** |
| --- | --- | --- | --- | --- | --- | --- |
| **BIOCARTA** | Map kinase inactivation of smrt corepressor | 0.0003 | Pertussis toxin-insensitive ccr5 signaling in macrophage | 2.64E-07* | Regulators of bone mineralization | 0.0004 |
|  | Alternative complement pathway | 0.0014 | Inhibition of matrix metalloproteinases | 1.11E-05* | Role of egf receptor transactivation by gpcrs in cardiac hypertrophy | 0.0029 |
| **KEGG** | Cell adhesion molecules (CAMs) | 1.72E-75* | Focal adhesion | 1.08E-31* | Protein digestion and absorption | 2.15E-56* |
|  | Vascular smooth muscle contraction | 3.17E-21* | Complement and coagulation cascades | 4.77E-24* | Renin-angiotensin system | 1.42E-07 |
|  | Malaria | 8.83E-10* | ECM-receptor interaction | 1.22E-20* | Bile secretion | 0.0038 |
|  | Axon guidance | 5.19E-08* | Vascular smooth muscle contraction | 6.42E-13* | Regulation of actin cytoskeleton | 0.0317 |
|  | Complement and coagulation cascades | 4.55E-07* | Osteoclast differentiation | 1.40E-09* | Amoebiasis | 0.0792 |
|  | Tight junction | 1.42E-06* | Prion diseases | 1.46E-09* | Circadian rhythm - mammal | 0.0852 |
|  | PPAR signaling pathway | 5.13E-05 | Endocytosis | 4.26E-07* | P53 signaling pathway | 0.1221 |
| **REACTOME** | BoNT Light Chain Types A, C1, E cleave SNAP-25 | 0* | Microtubule-dependent trafficking of connexons from Golgi to the plasma membrane | 0* | Amino acid transport across the plasma membrane | 0.0003 |
|  | Fructose catabolism | 0* | COX reactions | 0* | Peptide ligand-binding receptors | 0.0037 |
|  | Smooth Muscle Contraction | 5.24E-35* | Integrin cell surface interactions | 4.29E-25* | Cation-coupled Chloride cotransporters | 0.0087 |
|  | Transcriptional Regulation of White Adipocyte Differentiation | 1.93E-19* | Regulation of IGF Activity by IGFBP | 2.02E-20* | Hormone-sensitive lipase (HSL)-mediated triacylglycerol hydrolysis | 0.0129 |
|  | Interferon alpha/beta signaling | 1.61E-18* | Smooth Muscle Contraction | 4.02E-10* | Coenzyme A biosynthesis | 0.0145 |
|  | Nectin/Necl trans heterodimerization | 7.06E-07* | Activation of the AP-1 family of transcription factors | 7.10E-09* | Glycoprotein hormones | 0.0153 |
|  | cGMP effects | 4.34E-06* | Nitric oxide stimulates guanylate cyclase | 4.38E-08* | G beta:gamma signalling through PLC beta | 0.0217 |
|  | Interaction between L1 and Ankyrins | 2.88E-05* | Chemokine receptors bind chemokines | 9.43E-07* | Bicarbonate transporters | 0.0227 |
|  | Integrin cell surface interactions | 2.93E-05* | Interferon alpha/beta signaling | 8.76E-06* | Glutathione conjugation | 0.0227 |
| **NCI** | Syndecan-4-mediated signaling events | 0.0005 | Beta1 integrin cell surface interactions | 2.87E-21* | Beta1 integrin cell surface interactions | 0.0003 |
|  | Integrin-linked kinase signaling | 0.0028 | AP-1 transcription factor network | 1.84E-06* | Signaling mediated by p38-gamma and p38-delta | 0.0288 |
| **INOH** | Prostaglandin and Leukotriene metabolism | 2.63E-06* | Integrin signaling pathway | 5.08E-19* | Integrin signaling pathway | 4.06E-23* |
|  | BMP2 signaling pathway(through Smad) ( TGF-beta BMP Diagram) | 0.01307152 | IGF1 signaling pathway | 5.56E-09* | Heterotrimeric GPCR signaling pathway (through G alpha q, PLC beta and ERK cascade) | 1 |

* significant functions
