# Supplementary material for: Aberrant gene expression in mucosa adjacent to tumor reveals a molecular crosstalk in colon cancer
Source: Mol Cancer. 2014 Mar 5;13:46. doi: 10.1186/1476-4598-13-46 (PMC4023701; doi:10.1186/1476-4598-13-46)
Supplement: Additional file 8: Table S7 — Origin of proteins implicated in the crosstalk which are secreted by the tumor or located in tumor membrane. [file 1476-4598-13-46-S8.doc]

**Supplementary Table 7**: Cellular origin of proteins implicated in the crosstalk which are secreted by the tumor or located in tumor membrane.

|  | **epithelial** | **endothelial** | **fibroblasts** | **leukocytes** |
| --- | --- | --- | --- | --- |
| **SECRETED** | | | | |
| ADAM12 | 4.61133169 | 4.14087895 | 7.43043502 | 5.4477183 |
| AGT | 3.36793629 | 3.20881443 | 5.98189364 | 3.13297462 |
| APOC2 | 4.073567 | 4.13378722 | 4.25253283 | 4.20628218 |
| C3 | 3.64575296 | 3.33752431 | 8.94956246 | 3.39048035 |
| CFB | 4.90563587 | 3.6372882 | 6.11043838 | 3.24606714 |
| COL18A1 | 2.45818033 | 4.45881326 | 5.44864759 | 2.46983374 |
| COL1A1 | 4.33757841 | 5.7337503 | 11.4974336 | 4.2469831 |
| COL1A2 | 4.15446696 | 5.35336072 | 11.4166194 | 4.14202076 |
| COL3A1 | 3.99743709 | 7.05484669 | 12.0232324 | 3.87432814 |
| COL4A1 | 4.33637061 | 10.6244674 | 9.4072804 | 3.50258925 |
| COL4A2 | 2.97809274 | 8.75782432 | 7.60572129 | 2.4886943 |
| COL6A1 | 2.39978738 | 4.37396775 | 8.71657392 | 2.34739935 |
| COL6A3 | 4.95806304 | 5.67492269 | 11.500951 | 5.5363927 |
| CXCL5 | 1.78515149 | 1.48835134 | 3.18903304 | 1.72780992 |
| EFNA3 | 4.08664698 | 3.6284727 | 3.60831873 | 3.622472 |
| FN1 | 5.98736986 | 9.8426361 | 10.9967346 | 3.04593151 |
| IGHG1 | 1.93288468 | 2.85929463 | 1.98165048 | 4.00141514 |
| IL8 | 5.3219176 | 5.28210063 | 6.95766365 | 5.44245673 |
| INHBA | 3.96960848 | 3.54121603 | 8.34835562 | 3.32651064 |
| KAL1 | 4.56500582 | 4.88056734 | 7.88565376 | 5.08909691 |
| LAMA4 | 3.46923359 | 7.10957025 | 6.84780258 | 3.39193336 |
| MIF | 3.51561867 | 3.31807754 | 3.68665614 | 3.61523948 |
| MMP9 | 3.58960702 | 3.53898658 | 3.93959063 | 4.62913119 |
| PCSK9 | 4.70572765 | 3.7000294 | 3.74052722 | 3.78616664 |
| PF4 | 2.57910728 | 2.41479766 | 2.55791391 | 2.42326721 |
| PGF | 3.70815992 | 4.99928579 | 3.94572982 | 3.76917668 |
| PLA1A | 3.05865803 | 4.25660114 | 3.40903573 | 3.28132927 |
| PPBP | 2.96312253 | 2.33748929 | 2.29474987 | 2.4558952 |
| REG3A | 5.13966081 | 3.86844979 | 3.88652392 | 4.15918161 |
| SEMA3F | 4.83438513 | 8.74579466 | 4.48217381 | 3.86107824 |
| SFRP2 | 3.58673069 | 3.43912357 | 8.76491609 | 3.6498748 |
| SPP1 | 1.61354242 | 4.3581013 | 2.79602092 | 2.00313219 |
| TAC1 | 1.21732281 | 1.29457336 | 1.28995467 | 1.15617101 |
| TNC | 2.83794722 | 2.95196286 | 7.04489677 | 2.80070854 |
| VEGFA | 7.58927577 | 5.36830803 | 7.3443709 | 4.82234959 |
| LIF | 3.19950139 | 2.64695034 | 3.8785475 | 3.33174073 |
| WNT5A | 2.06899778 | 2.07109099 | 6.38538957 | 2.08172031 |
| ELN | 3.72355376 | 5.05891788 | 7.12758057 | 3.61448424 |
| CCL2 | 3.85237184 | 6.71830445 | 8.05893649 | 4.14849348 |
| **MEMBRANE** | | | | |
|  | **epithelial** | **endothelial** | **fibroblasts** | **leukocytes** |
| CD81 | 6.46002117 | 8.24826325 | 7.77103156 | 6.0269912 |
| FGFR4 | 5.09716277 | 3.81989205 | 4.32327355 | 3.34413131 |
| ITGA1 | 2.62721456 | 3.93202745 | 4.72255915 | 3.53208493 |
| LRP8 | 4.3559145 | 3.76682143 | 3.14343445 | 4.24295906 |
| MME | 3.92108017 | 3.61902152 | 4.02227825 | 3.78142442 |
| MMP14 | 4.44439733 | 4.56547129 | 5.22083878 | 4.21443397 |
| NEDD4 | 2.66939633 | 2.69248286 | 2.55289134 | 2.88492947 |
| ROBO1 | 3.00835442 | 5.46317744 | 7.16759861 | 2.97615491 |
| ROBO2 | 4.20380095 | 4.241713 | 5.03047215 | 4.24334223 |
| ROBO4 | 2.30480774 | 7.24028186 | 2.16676178 | 1.99086166 |
| SDC3 | 5.38271665 | 5.89651399 | 6.49545323 | 4.27282515 |
| IGDCC4 | 1.56247207 | 1.26742455 | 4.6761279 | 1.22431502 |
| SELE | 2.89527087 | 7.64113993 | 2.70457049 | 3.17527126 |
| CD82 | 3.54747048 | 3.36125588 | 3.68392974 | 3.82169632 |
| CXCR4 | 3.78170262 | 6.56070195 | 6.15165239 | 10.7007284 |

* In orange, cell type specific over-expression.

** In yellow, over-expression shared by two cell types.

*** In red, bad quality hybridization probe.
