# Supplementary material for: Aberrant gene expression in mucosa adjacent to tumor reveals a molecular crosstalk in colon cancer
Source: Mol Cancer. 2014 Mar 5;13:46. doi: 10.1186/1476-4598-13-46 (PMC4023701; doi:10.1186/1476-4598-13-46)
Supplement: Additional file 9: Table S8 — Baseline characteristics of healthy donors and CRC patients. [file 1476-4598-13-46-S9.doc]

Supplementary Table 8. Baseline characteristics of healthy donors and CRC patients.

| **Healthy donors (n=50)** |  |
| --- | --- |
| *Gender* |  |
| Male | 27 (54%) |
| Female | 23 (46%) |
| *Median age (range,years)* | 63 (25-88) |
| *Site* |  |
| Right | 27 (54%) |
| Left | 23 (46%) |
| **Cases (n=98)** |  |
| *Gender* |  |
| Male | 71 (72.4%) |
| Female | 27 (27.6%) |
| *Median age (range,years)* | 71 (43 - 87) |
| *Site* |  |
| Right | 38 (38.8%) |
| Left | 60 (61.2%) |
| *Stage* |  |
| II A | 90 (91.8%) |
| II B | 8 (8.2%) |
| *Recurrence* |  |
| No relapse | 76 (77.6%) |
| Relapse | 22(22.4%) |
| *Recurrence-free median time (range,months)* | 67.8 (24.8 – 136.9) |
